# Development and Validation of a Brief Self-Report Scale to Measure Perceived Immune Vulnerability (PIV) in Cancer Population

**DOI:** 10.3390/cancers17223713

**Published:** 2025-11-20

**Authors:** Lingyun Sun, Rose Wai-Yee Fok, Jerrin Bawa, Xiaotong Li, Kaitlin Lampson, Susan Chimonas, Thomas M. Atkinson, Jun J. Mao

**Affiliations:** 1 Oncology Department, Xiyuan Hospital, China Academy of Chinese Medical Sciences, Beijing 100091, China; 2 Integrative Medicine Service, Department of Medicine, Memorial Sloan Kettering Cancer Center, New York, NY 10065, USA; 3 Division of Medical Oncology, National Cancer Centre Singapore, Singapore 168583, Singapore; 4 Epidemiology-Biostatistics, Memorial Sloan Kettering Cancer Center, New York, NY 10065, USA; 5 Psychiatry and Behavioural Sciences, Memorial Sloan Kettering Cancer Center, New York, NY 10065, USA

**Keywords:** perceived immune vulnerability, cancer, patient-reported outcome, scale development and validation

## Abstract

Objective: Understanding immunity from the patient’s perspective is essential for optimizing cancer treatment and improving patient-reported outcomes. Currently, there are no established self-reported scales designed to assess immune vulnerability in cancer populations. We aimed to fill this gap by developing a brief scale fit for this purpose. Methods: We developed a five-item brief perceived immune vulnerability (PIV) scale and conducted a two-phase study on cancer survivors in China. In Phase 1, we assessed the reliability and factor structure of the PIV scale, while in Phase 2, we evaluated its construct validity by comparing it with measures of overall well-being and various patient characteristics. Results: Between October and December 2022, 100 patients completed Phase 1, while 1275 patients were enrolled in Phase 2. The mean age of the participants was 68.7 years (SD 10.3); 892 (64.9%) patients were female, and 537 (40.3%) patients had at least high-school education. In Phase 1, Cronbach’s α = 0.913, indicating good internal consistency. We identified one component with an eigenvalue of 3.72, explaining 70.17% of the variance. In Phase 2, patients reporting poor well-being had worse immune function (Pearson *r* = 0.202, *p* < 0.001). Additionally, patients undergoing active cancer treatment had worse immune function than those receiving survivorship care (*p* = 0.024). Meanwhile, patients with stage III/IV disease had worse immune function than those with stage I/II disease (*p* = 0.041). Conclusions: The PIV scale is a reliable and valid tool for assessing immune vulnerability in patients with cancer and preliminarily offers a convenient approach to monitoring and evaluating their self-reported immunity. Further research is needed to explore the broader utility of this instrument.

## Simple Summary

The aim of this study was to develop a simple tool to capture how cancer patients perceive their own immune vulnerability, an important but previously unmeasured aspect of patient experience. The researchers created the five-item perceived immune vulnerability (PIV) scale and evaluated it in a two-phase study involving cancer survivors in China. In the first phase, they examined the scale’s reliability and underlying structure, confirming that it functions consistently as a brief, unified measure. In the second phase, they tested how well the scale reflects related aspects of patient health and characteristics. Patients who reported poorer well-being or were in more intensive stages of care tended to perceive greater immune vulnerability, supporting the scale’s validity. Overall, the PIV scale appears to be a reliable, practical tool for assessing patients’ self-reported immunity and may help clinicians better understand and monitor immune-related concerns in cancer care. Further study is encouraged to expand its use.

## 1. Introduction

Immunity refers to the body’s ability to defend itself against harmful or toxic substances by identifying and eliminating potentially dangerous pathogens such as bacteria, viruses, and cancer cells [1]. Immunity is highly relevant to cancer patients, as cancer and its treatments can weaken the immune system. Immunotherapy, which enhances the body’s immune system to allow it to fight cancer, is an emerging and effective treatment modality [2]. Moreover, a strong immune system can improve responses to cancer medications, aid in recovery, help patients overcome infections, and reduce complications during treatment [3]. Recently, the impact of infectious diseases such as COVID-19, which compromises the immune system, has become a significant concern, as cancer patients are at high risk of developing COVID-19-related complications [4]. Therefore, maintaining and supporting a healthy immune system constitute a critical component of cancer management and improving patient outcomes [5,6].

Monitoring immunity in cancer patients is essential for guiding physicians’ care decisions. However, current objective immunity measurements have several limitations. Conventional methods, such as complete blood counts, antibody testing, cytokine testing, and immune cell analysis, are invasive, costly, and time-consuming [7]. Furthermore, the laboratory results they yield reflect only a patient’s immune function at the time of testing and fail to account for their perception of their immune status, thereby overlooking a crucial aspect of the patient’s experience and motivations for seeking care [8,9]. As a result, there is no universally accepted gold standard for evaluating immune function in clinical practice, especially for the cancer population.

Patient-reported outcomes (PROs) are a commonly used means of assessing a patient’s subjective health status [10]. Two existing immune function scales incorporate symptoms such as cough, headache, and diarrhea as surrogate markers to assess immunity in the general population [11,12]. However, in cancer patients, these symptoms may reflect underlying adverse events and toxicities related to cancer or its treatment rather than the patient’s baseline immune function [13,14]. Based on the theory of the behavioral immune system [15], the 15-item Perceived Vulnerability to Disease Questionnaire (PVD) was developed and has been widely used to measure patients’ beliefs about personal susceptibility to infectious disease [16]. To date, this scale has not been used to measure immune responses in the cancer population.

To address this gap, we aimed to develop a 5-item brief perceived immune vulnerability (PIV) scale, a PRO measure, to evaluate perceived immune function among patients with cancer and to perform an initial assessment of its reliability and validity in this two-phase study. We hypothesized that the PIV scale would be feasible for use and reliable in clinical cancer care settings. Additionally, we hypothesized that patients undergoing active cancer treatments and with advanced disease or poorer well-being would report worse perceived immune vulnerability [17,18].

## 2. Methods

### 2.1. Study Population

We conducted both Phase 1 and Phase 2 studies on Chinese cancer survivors who had consumed an herbal supplement (Reishi) produced by the company Zhongke Health International LLC between October 2022 and December 2022 in China. Convenience samples were sequentially collected through the company’s consumers’ database, covering 22 provinces around China. The commercial company involved in patient recruitment and data collection provided logistical support only and had no role in the study’s design, data management, statistical analysis, interpretation of findings, manuscript preparation, or publication decisions. The research team retained full independence throughout all phases of the study. The partial funding received did not influence the study’s conduct or outcomes.

As per the inclusion criteria, we included patients aged between 18 and 80 years with a confirmed pathological diagnosis of malignant disease. Informed written consent was obtained from all participants. As a token of appreciation, a gift (equal to CNY 50) was provided to those who completed the entire survey study. This study was approved by the Ethics Committee of Xiyuan Hospital of the China Academy of Chinese Medical Sciences (2022-XLA129-1) on 19 August 2022. Written informed consent was obtained from all participants before enrollment.

### 2.2. Instrument Development

The three authors (JJM, LYS, and XTL) developed the original items of the PIV scale following a review of the existing literature on PROs regarding immunity and further discussion [19,20,21]. We adopted the definition used in biology, which states that immunity is the state of being insusceptible or resistant to a noxious agent or process, especially a pathogen or infectious disease [22]. The common cold (a viral infection of the upper respiratory tract, primarily affecting the nose, throat, and sinuses) is one of the most prevalent infectious diseases in the general population, estimated to occur in 2 to 4 adults per year [23,24]. A survey study conducted on middle-aged and elderly healthy Japanese people showed that the prevalence of a self-reported predisposition to common cold was 2.1% [25]. Cancer patients are even more susceptible to the common cold because their immune systems are already weakened by cancer or its treatments. Thus, based on this concept together with epidemiologic evidence, our study team chose to use the common cold to illustrate immunity.

We developed four questions that asked patients about their susceptibility to and the frequency, severity, and duration of cold symptoms over the past three months [26]: ‘I catch colds easily’, ‘I catch colds more frequently than other people’, ‘If I catch a cold, my symptoms are more severe than those of other people’, and ‘If I catch a cold, my illness duration is longer than that of other people.’ These items were evaluated using a 5-point Likert scale (1 = Strongly Disagree, 2 = Disagree, 3 = Not Sure, 4 = Mostly Agree, and 5 = Completely Agree). Additionally, we included a question requiring patients to self-evaluate their overall immunity over the past three months: ‘During the past three months, how would you evaluate your immunity? (1 = Very bad, 2 = Bad, 3 = Average, 4 = Good, and 5 = Very good).’ Then, for statistical analysis, we reversed the score for this question, with 1 indicating very good overall immunity and 5 indicating very poor immunity. We used a 3-month period in the scale to reduce patient recall bias. The cumulative score of all five items represents the patient’s self-assessed immunity level, ranging from 5 to 25. A higher score indicates a poorer patient-perceived immune function level. In September 2022, we conducted a pilot survey with 20 cancer survivors in Beijing, China, gathering their qualitative feedback on the content and clarity of the scale. All participants found the PIV scale easy to understand and complete. As a result, no items were modified or removed following the pilot study.

### 2.3. Study Administration

Edmonton Symptom Assessment Scale (ESAS) was used to evaluate the symptom burden and overall well-being of the cancer patients. During statistical analysis phase, we only used the item “well-being” to evaluate its association with PIV scale, which ranged from 0 (best well-being) to 10 (worst well-being) [27]. ESAS well-being was defined as “good” (0–3), “moderate” (4–6), or “bad” (7–10) according to cut-offs reported in the existing literature [28]. Patients’ demographic characteristics and cancer-related information, including diagnosis, current treatment status, and disease stage, were collected through patient self-reports. All surveys were distributed and completed via an online questionnaire system (Tencent Online Survey).

### 2.4. Statistical Analysis

We used descriptive statistics to examine missing data and item distribution. We performed principal component factor (PCF) analyses to identify the factor structure of the scale. The number of factors was determined via examination of eigenvalues ≥ 1.00. Confirmatory factor analysis (CFA) was performed to assess the construct validity of the scale. Cronbach’s α statistics were calculated to determine the internal consistency of the scale. Coefficients of 0.70 or greater are considered to be acceptable for an instrument developed to evaluate differences in group means [29]. Normal distribution of the PIV scale total score was by using the Z-score (skewness value/standard error of skewness). If z-scores of skewness and kurtosis were smaller than 1.96 (for %5 of the type I error rate), the data were considered normal [30]. Interquartile range (IQR) was calculated for the PIV scale total score and each item. To evaluate the known-group validity, we compared the PIV scale scores between patients of different cancer stages, cancer treatment statuses, and levels of overall well-being. Data analysis was performed using SPSS 26.0 for macOS (IBM SPSS Statistics). All analyses were 2-sided, and a *p*-value < 0.05 was considered to be statistically significant.

## 3. Results

### 3.1. Patients’ Characteristics

Out of the 1600 cancer patients and survivors we approached, 1375 (85.9%) agreed to participate and completed the survey. The mean age of all the participants was 68.7 (SD 10.3). A total of 892 (64.9%) of the patients were female, and 537 (40.3%) of the patients had an above-high-school level education. The vast majority of the participants were retired (*n* = 1284, 93.4%). The average number of years since cancer diagnosis was 8.5 (SD 6.2). The most common cancer types were breast (*n* = 373, 27.1%), gastrointestinal (356, 25.9%), and lung (*n* = 276, 20.1%). Most patients had a current diagnosis of stage I-III disease, with only 82 (8.1%) having a diagnosis of stage IV disease. A total of 181 (15.8%) patients were receiving active cancer treatment, and 967 (84.2%) were cancer survivors. A total of 136 patients with stage IV disease were undergoing active cancer treatment, 35 (25.7%) were receiving chemo-/radio-therapy, and 101 (74.3%) were receiving targeted therapy or immunotherapy (Table 1).

### 3.2. Items Distribution

**Figure 1:**
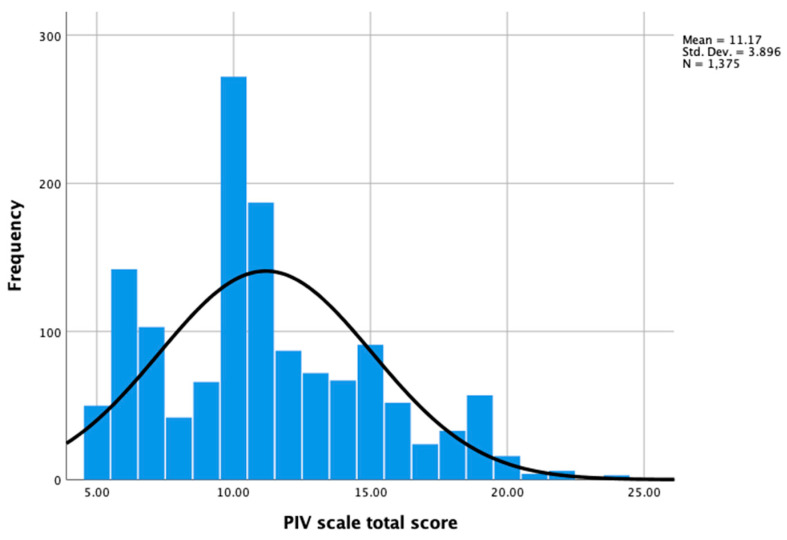
Distribution of PIV scale total scores (*n* = 1375).

The distributions of all the participants’ responses (*n* = 1375) regarding the total score and five items in the PIV scale are shown in Figure 1 and Table 2, respectively. The interquartile range (IQR) was 5 for the total score for the PIV scale (the Z-score was 8.5, indicating a non-normal distribution). No item had over 5% missing data. Floor effects were found for items 2–5. Overall, 27.1%, 28.4%, 24.0%, and 23.6% of patients reported “Not at all agree” for the statements in items 2–5, respectively.

### 3.3. Reliability and Factor Analysis

In Phase 1, the five-item PIV scale showed good internal consistency, with a Cronbach’s α of 0.913 and a median (IQR) score of 10 (6). In a principal component factor analysis (varimax rotation) conducted to explore the factor structure of the scale in the phase 1 study, we identified and retained one component with an eigenvalue of 3.72, explaining 70.17% of the variance. No items were removed from or added to the original scale based on these results.

### 3.4. Construct Validity and Known-Group Validity

**Figure 2:**
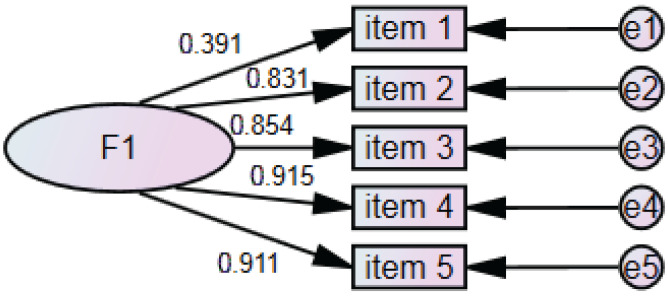
Confirmatory factor analysis on PIV scale in phase 2 study.

In the phase 2 study, the median (IQR) total score of the PIV scale was 11 (5), with a Cronbach’s α of 0.888. We used the method of modified residuals for model correction (e2-e3MI = 427.736). Confirmatory factor analysis was conducted to examine the factors and construct validity of the scale. After correction, the X^2^/DF was below 5, the RMR and RMSEA were below 0.08, and the CFI, TLI, IFI, and GFI were all above 0.9. From this, it can be seen that the PIV scale has good construct validity. The factor loadings of each item on the PIV scale are all greater than 0.35, indicating that the items are highly representative of their respective latent variables. Additionally, the average variance extracted (AVE) for each latent variable is greater than 0.5, and the composite reliability (CR) exceeds 0.7, indicating good convergent validity (Figure 2).

**Figure 3:**
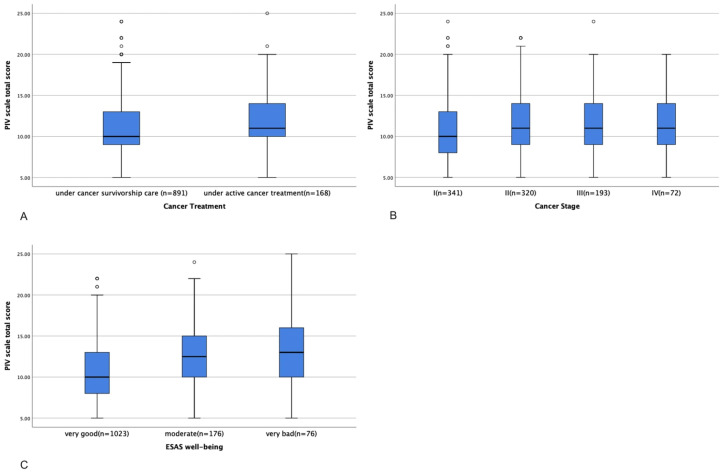
Differences in PIV scale total score between patients with different treatment statuses (**A**), cancer stages (**B**), and ESAS levels of well-being (**C**).

For the PIV scale, the IQR was 5, and the Z-score was 7.7, indicating a non-normal distribution. Thus, Kruskal–Wallis tests were used to analyze the differences in the total scores of the PIV scale between groups. Patients undergoing active cancer treatment had significantly higher PIV scale total scores (indicating a worse immune function status) than patients in survivorship care (11.76 ± 3.71 vs.11.04 ± 3.76, *p* = 0.012, Figure 3A). Patients with stage II (11.41 ± 3.95), III (11.49 ± 3.84), and IV (11.46 ± 3.89) cancer had significantly higher PIV scale total scores than patients with stage I disease (10.69 ± 3.82, *p* = 0.038, Figure 3B). Patients who reported very good (10.83 ± 3.73) and moderate (12.68 ± 3.85) well-being had significantly lower PIV scale total scores than those who reported poor well-being (12.67 ± 4.41, *p* < 0.001, Figure 3C), indicating a better immune function status.

## 4. Discussion

This study provides preliminary evidence that the novel five-item brief perceived immune vulnerability (PIV) scale is a reliable and valid PRO measure of self-reported immunity for patients with cancer. The PIV scale demonstrated good internal consistency and sufficient sensitivity for detecting differences based on patients’ overall well-being, treatment status, and cancer stage. These findings support our hypothesis that the PIV scale may be a feasible and practical tool for measuring the immune function of patients with cancer.

Compared to existing questionnaires assessing immune status, the PIV scale offers several distinct advantages. First, it provides a concise, simple, and user-friendly approach by focusing on a single key infectious disease—the common cold—as an indicator of immune function (thus contrasting with the 19-item Immune Function Questionnaire [11] and 7-item Immune Status Scale [12]). Second, unlike traditional questionnaires that primarily assess symptom occurrence, the PIV scale incorporates measures of susceptibility, severity, and duration, enhancing its internal consistency and reliability. Another key difference is that our scale assesses immune status over a three-month period, whereas previous questionnaires evaluate a 12-month timeframe, making it more challenging to track clinical changes over shorter durations. Finally, to our knowledge, the PIV scale is the only PRO measure of perceived immune vulnerability specifically developed for and validated using cancer patients, highlighting its novelty and relevance for this population.

In this study, we further validated the PIV scale by demonstrating its ability to distinguish perceived immune vulnerability among patients at different cancer stages and with different treatment statuses. Cancer patients are particularly susceptible to infections due to chemotherapy-induced immunosuppression and the burden of the tumor itself [31]. In line with this, our study found that patients undergoing active cancer treatment and those with advanced-stage cancer reported poorer self-evaluated immune function. Additionally, factors such as stress, nutrition, and age have been shown to influence immune function [32,33,34]. Our findings further demonstrate that patients with better overall well-being reported higher self-perceived immune function. However, we did not observe a significant association between age and PIV scale scores in our study population. Future research could explore the impact of potential factors on immune status to foster a more comprehensive understanding of patient-reported immune function.

This novel perceived immune vulnerability scale is not intended to replace objective laboratory-based immune assessments. Instead, it complements these measures by providing a broader perspective on immune function by assessing patient perception. Conventional immune function tests, such as whole blood cell counts, cytokine analysis, and antibody measurements, are valuable for specific diseases and treatment conditions. However, they are invasive, costly, and time-consuming [35]. Moreover, these tests capture only a single point in time, failing to reflect long-term immune function trends or patients’ lived experiences. In contrast, the PIV scale offers a more dynamic and personalized assessment of perceived immune vulnerability over time. The self-reported measures can also capture subjective experiences, such as susceptibility to the common cold, the duration and severity of illness, and overall well-being. Combining patient-reported outcomes with objective immune markers could enhance clinical decision-making and facilitate a more comprehensive evaluation of immune health. For instance, many patients seek support through integrative oncology approaches—including exercise, dietary interventions, mind–body therapy, acupuncture, and supplements—to boost immunity [36,37,38,39]. Our instrument could be a reliable tool allowing both patients and health care providers to efficiently monitor and evaluate perceived immune vulnerability during survivorship care or active cancer treatments.

This study has several limitations. First, since the research was conducted exclusively in China, the applicability of the findings to cancer populations outside this context requires further validation. Second, the study population was limited to cancer patients who took Reishi, so their self-reported immune vulnerability could be impacted by their supplement intake. In the future, our research team will validate this scale in a broader cancer population and in healthy populations not only in China but also in other countries after translating it into the corresponding languages. Third, logistical constraints prevented us from performing a retest survey in both the Phase 1 and Phase 2 studies, hampering our ability to assess the test–retest reliability of the scale. Fourth, the lack of prospective data in the current study prevents the establishment of clinically meaningful score changes for the PIV scale. In addition, this study was carried out during a peak period of COVID-19, which could have adversely affected overall immune function in the phase 2 study compared to the phase 1 study. Finally, we did not use established objective standards for immune function, such as lymphocyte subsets or other widely recognized questionnaires, to test the criterion validity of the scale. While these measures are not definitive indicators of immune function in cancer patients, future research should explore the relationship between laboratory tests and the PIV scale to better determine the validity of its criteria.

## 5. Conclusions

In conclusion, this study provides initial evidence that the PIV scale is a reliable and valid tool for assessing perceived immune vulnerability in cancer patients. However, further research is needed in order to explore additional aspects of its reliability and validity, such as test–retest reliability and sensitivity to change, through longitudinal studies. Ultimately, this instrument has the potential to enhance the monitoring and evaluation of immunity in cancer patients during survivorship and treatment, offering a more convenient and effective means of outcome measurement and healthcare assessment.

## Figures and Tables

**Table 1:** Patients’ characteristics.

		Combined		Phase 1		Phase 2	
Characteristics		N = 1375	%	N = 100	%	N = 1275	%
Age	≤65	438	31.9	46	46.0	392	30.7
	>65	937	68.1	54	54.0	883	69.3
Gender	Male	483	35.1	32	32.0	451	35.4
	Female	892	64.9	68	68.0	824	64.6
Education	≤High school	794	59.7	62	63.3	732	59.4
	>High school	537	40.3	36	36.7	501	39.3
Work status	Working	91	6.6	8	8.0	83	6.5
	Retired	1284	93.4	92	92.0	1192	93.5
Year since cancer diagnose	≤1 year	75	5.5	8	8.0	67	5.3
	1–5 years	387	28.1	37	37.0	350	27.5
	≥5 years	913	66.4	55	55.0	858	67.3
Cancer type	Lung	276	20.1	26	26.0	250	19.6
	GI	356	25.9	20	20.0	336	26.4
	Breast	373	27.1	24	24.0	349	27.4
	Gynecologic	109	7.9	9	9.0	100	7.8
	Other	261	19.0	21	21.0	240	18.8
Cancer stage	I	380	37.5	39	44.8	341	36.8
	II	338	33.4	18	20.7	320	34.6
	III	213	21.0	20	23.0	193	20.8
	IV	82	8.1	10	11.5	72	7.8
Treatment status *	Active treatment	181	15.8	76	85.4	891	84.1
	Survivorship care	967	84.2	13	14.5	168	15.9
Advanced cancer treatment (*n* = 181) **	Chemo-/radiotherapy	35	25.7	3	42.9	32	24.8
	Targeted therapy	47	34.6	3	42.9	44	34.1
	Immunotherapy	54	39.7	1	14.3	53	41.1

* Missing and unknown data existed for this variable; the total number did not sum to 1375. ** Missing and unknown data existed for this variable; the total number did not sum to 181.

**Table 2:** Distribution of patients’ responses for each item of the PIV scale (*n* = 1375).

		IQR	Mean(SD) (Range 1–5)	Very Good %	Good %	Average %	Bad %	Very Bad %
Item 1	How would you evaluate your immunity over the past three months?	1.00	2.5(0.80)	7.4	41.7	41.4	7.9	1.5
	**Please rate the following statements**			**Strongly Agree** **%**	**Disagree** **%**	**Not Sure** **%**	**Mostly Agree** **%**	**Completely Agree** **%**
Item 2	I catch colds easily	2.00	2.14(0.98)	27.1	44.7	16.6	10.0	1.5
Item 3	I catch colds more frequently than other people.	1.00	2.07(0.93)	28.4	47.2	14.8	8.6	1.1
Item 4	If I catch a cold, my symptoms are more severe than other people’s.	1.00	2.18(0.96)	24.0	46.7	17.5	10.6	1.2
Item 5	If I catch a cold, my illness lasts longer than in other people.	1.00	2.24(0.99)	23.6	44.3	18.3	12.4	1.5

PIV, perceived immune vulnerability. IQR, Interquartile Range.

## Data Availability

The data that support the findings of this study are available from the corresponding author, Jun J. Mao, upon reasonable request.

## Abbreviations

PIV	Perceived immune vulnerability
PRO	Patient-reported outcome
ESAS	Edmonton Symptom Assessment Scale
PCF	Principal component factor
IQR	Interquartile range
SD	Standard deviation
